# Integrating Multiple Inputs Into an Artificial Pancreas System: Narrative Literature Review

**DOI:** 10.2196/28861

**Published:** 2022-02-24

**Authors:** Chirath Hettiarachchi, Elena Daskalaki, Jane Desborough, Christopher J Nolan, David O’Neal, Hanna Suominen

**Affiliations:** 1 School of Computing College of Engineering and Computer Science The Australian National University Canberra Australia; 2 Department of Health Services Research and Policy, Research School of Population Health College of Health and Medicine The Australian National University Canberra Australia; 3 Australian National University Medical School College of Health and Medicine The Australian National University Canberra Australia; 4 John Curtin School of Medical Research College of Health and Medicine The Australian National University Canberra Australia; 5 Department of Medicine University of Melbourne Melbourne Australia; 6 Department of Endocrinology and Diabetes St Vincent’s Hospital Melbourne Melbourne Australia; 7 Data61 Commonwealth Industrial and Scientific Research Organisation Canberra Australia; 8 Department of Computing University of Turku Turku Finland

**Keywords:** diabetes mellitus, type 1, pancreas, artificial, algorithms, multivariate analysis, insulin infusion systems, control systems

## Abstract

**Background:**

Type 1 diabetes (T1D) is a chronic autoimmune disease in which a deficiency in insulin production impairs the glucose homeostasis of the body. Continuous subcutaneous infusion of insulin is a commonly used treatment method. Artificial pancreas systems (APS) use continuous glucose level monitoring and continuous subcutaneous infusion of insulin in a closed-loop mode incorporating a controller (or control algorithm). However, the operation of APS is challenging because of complexities arising during meals, exercise, stress, sleep, illnesses, glucose sensing and insulin action delays, and the cognitive burden. To overcome these challenges, options to augment APS through integration of additional inputs, creating multi-input APS (MAPS), are being investigated.

**Objective:**

The aim of this survey is to identify and analyze input data, control architectures, and validation methods of MAPS to better understand the complexities and current state of such systems. This is expected to be valuable in developing improved systems to enhance the quality of life of people with T1D.

**Methods:**

A literature survey was conducted using the Scopus, PubMed, and IEEE Xplore databases for the period January 1, 2005, to February 10, 2020. On the basis of the search criteria, 1092 articles were initially shortlisted, of which 11 (1.01%) were selected for an in-depth narrative analysis. In addition, 6 clinical studies associated with the selected studies were also analyzed.

**Results:**

Signals such as heart rate, accelerometer readings, energy expenditure, and galvanic skin response captured by wearable devices were the most frequently used additional inputs. The use of invasive (blood or other body fluid analytes) inputs such as lactate and adrenaline were also simulated. These inputs were incorporated to switch the mode of the controller through activity detection, directly incorporated for decision-making and for the development of intermediate modules for the controller. The validation of the MAPS was carried out through the use of simulators based on different physiological models and clinical trials.

**Conclusions:**

The integration of additional physiological signals with continuous glucose level monitoring has the potential to optimize glucose control in people with T1D through addressing the identified limitations of APS. Most of the identified additional inputs are related to wearable devices. The rapid growth in wearable technologies can be seen as a key motivator regarding MAPS. However, it is important to further evaluate the practical complexities and psychosocial aspects associated with such systems in real life.

## Introduction

### Background

In health, pancreatic islet β-cells respond to metabolic and neurohormonal signals to secrete insulin into the portal vein at finely controlled variable rates to ensure that blood glucose level and overall metabolic homeostasis are maintained. Diabetes is a metabolic disease characterized by elevated blood glucose concentrations as a consequence of an absolute deficiency of insulin secretion or inadequate insulin secretion to compensate for ineffective insulin action. Type 1 diabetes (T1D) is caused by the autoimmune destruction of the islet β-cells and results in absolute insulin deficiency [1]. An inability to match insulin delivery with an individual’s changing insulin requirements results in either hypoglycemia (low blood glucose level) or hyperglycemia (high blood glucose level). Hypoglycemia, if severe, may result in loss of consciousness, seizures, or even death. Long-term exposure to hyperglycemia results in complications such as blindness, limb amputations, and cardiovascular disease. Maintaining blood glucose levels in a healthy range is essential for the avoidance of severe short- and long-term complications of diabetes [1].

The discovery and use of exogenous insulin administration since 1921 as a therapeutic agent has been life saving for people living with T1D. More recently, pancreas and islet cell transplants have also provided a solution for T1D, although organ donation shortage, the risks of surgery, and the need for immunosuppression are limiting factors [2]. As a result, there is a continued reliance on the subcutaneous administration of exogenous insulin to treat this condition. There have been continuous advancements in insulin preparations [3], insulin delivery [4], and blood glucose level monitoring [5]. Until recent years, best practice treatment of T1D, as was established in the Diabetes Control and Complications Trial [6], involved frequent self-monitoring of blood glucose level through using finger pricks to access capillary blood and multiple daily injections of short- and long-acting insulins. Information from the self-monitoring of blood glucose level as well as the carbohydrate content of meals and planned exercise informed the titration of insulin doses. The advent of rapid-acting insulin analogs, continuous glucose monitoring (CGM), continuous subcutaneous infusion of insulin (CSII), shortcomings in manual insulin-dose determination, and the significant psychological burden [7] have motivated the development of the artificial pancreas (AP; or AP systems [APS]) [8].

**Figure 1:**
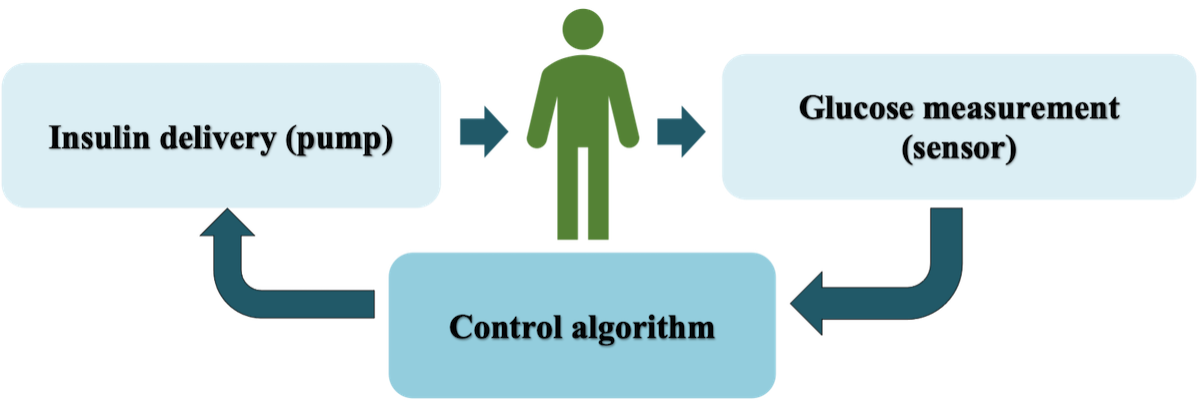
The basic system architecture of the artificial pancreas.

Although the concept of the AP has been around for >40 years, with the Biostator [9] identified as the first closed-loop glucose controlling system or AP [10], it is only in the last few years that the use of the AP has become a clinical reality. The first Food and Drug Administration (FDA)–approved commercial AP was released in 2016 in the United States, with a second system more recently approved [11,12]. The basic components of the APS are a sensor measuring subcutaneous interstitial fluid glucose on a near-continuous basis, a pump infusing rapid-acting insulin into the subcutaneous tissue, and a control algorithm (also known as the controller) that uses glucose measurements as the main input to calculate and operate the required rate of insulin infusion as the output (Figure 1). Proportional integral derivative control, model predictive control (MPC), fuzzy logic [13-15], adaptive control [16,17], and reinforcement learning [18] have been used in the recent past for controller development. The FDA has categorized the AP as a class III medical device, which is considered high risk. Hence, an investigation device exemption is required before conducting a clinical trial [19]. This requires initial testing of the proof of concept through animal trials, which is a time-consuming and costly exercise. A critical step toward AP advancement was the development of physiological models and simulators, which enabled the tuning and testing of different control algorithms in silico before conducting clinical studies, ensuring safety. The minimal model of glucose kinetics [20], the Sorenson model [21], the Hovorka model [22], the UVA/PADOVA simulator [23], the mGIPsim simulator [24], and the in silico patient population by Resalat et al [25] are some of the widely used models. The UVA/PADOVA simulator is currently the only FDA-approved simulator.

The major challenges with respect to the APS control algorithms relate to (1) delays in the onset and offset of insulin action because of delays of its absorption from subcutaneous depots (from CSII delivery) into the blood and (2) a time lag between glucose levels measured in subcutaneous interstitial fluid and blood glucose levels measured by currently available CGM devices. These limitations of APS imposed by the pharmacokinetics of subcutaneously delivered insulin and measured glucose levels are most evident in situations in which blood glucose levels and insulin requirements change rapidly and unexpectedly. These include meals, exercise, stressful events, and in response to acute illnesses. The current APS are hybrid closed-loop systems that require user input regarding meals and exercise; hence, similar to previous treatment methods, there remains a cognitive burden, affecting the quality of life of people with T1D [26]. Despite these limitations, systematic reviews and meta-analyses have verified that APS have shown better performance than conventional pump therapy [27]. However, there is still significant room for improvement.

Approaches used for improving APS functionality include advances in CGM accuracy and reliability; the development of faster-acting insulin analogs; and dual hormone infusion systems [28] in which glucagon, which can prevent hypoglycemia, as well as insulin can be delivered independently through the use of a controller. Complications of T1D can be related to meals, exercise, stress, and illness, all of which may affect glucose homeostasis. Current systems are unable to recognize these events and rely almost entirely upon inputs based on glucose level measurements and a record of the amount of insulin delivered. Inputs in addition to glucose level measurements may overcome some of the limitations of the current-generation APS. There has been recent focus on integrating additional external inputs captured from wearable devices and invasive sensors as part of experimental multi-input APS (MAPS). The addition of various signal inputs (eg, lactate and heart rate [HR]) is expected to provide more information and support the automatic identification of activities such as meals, exercise, sleep, stress, and other biological variations that affect the glucose profile [29]. The early detection of these activities would also help to counter limitations arising from CGM sensor delays [30]. This is also expected to reduce cognitive burden through lessening user interaction, leading to a better quality of life [31]. The rapid development of wearable sensor technologies can be identified as a strong motivator with respect to MAPS; however, it is important to analyze the potential improvement and additional device burden arising through the use of these systems.

### Objectives

The main objective of this survey is to identify and review the MAPS that have been proposed to date in terms of used inputs, control architectures, and validation methods. To develop better systems, it is critical to understand the current state of MAPS and identify associated complexities. We aim to achieve this through conducting an in-depth analysis of previous related studies. The current pace of APS development has been slow, prompting movements such as #WeAreNotWaiting by people with T1D, which focuses on do-it-yourself APS [32]. This synthesis may accelerate work on developing improved MAPS. This survey identified a variety of additional signals that have been integrated into experimental APS. Most of the reviewed publications focused upon noninvasive inputs from wearable devices. These additional input signals have been integrated into different architectures to augment the controllers, in particular (1) for activity detection and switching of controller modes, (2) as direct inputs to the controller for decision-making, and (3) for the development of intermediate modules of the controller (eg, hypoglycemia prediction or meal detection). A variety of physiological models, simulation environments, and clinical studies have been used for validation of the results. A detailed analysis is presented in later sections.

## Methods

### Overview

The survey was conducted by 3 independent reviewers (CH, ED, and HS) with research backgrounds in engineering, signal processing, machine learning, and health informatics, supported by a research librarian. The first reviewer conducted a systematic literature search and shortlisted studies through title and abstract screening. The second and third reviewers provided input to select the final studies for the survey and conducted the analysis. Throughout the reviewing process the researchers obtained valuable clinical expertise from 2 endocrinologists actively involved in T1D management and lived experience insights from young people with T1D within the Health Experience Team of the Our Health in Our Hands [33] strategic initiative of the Australian National University.

**Figure 2:**
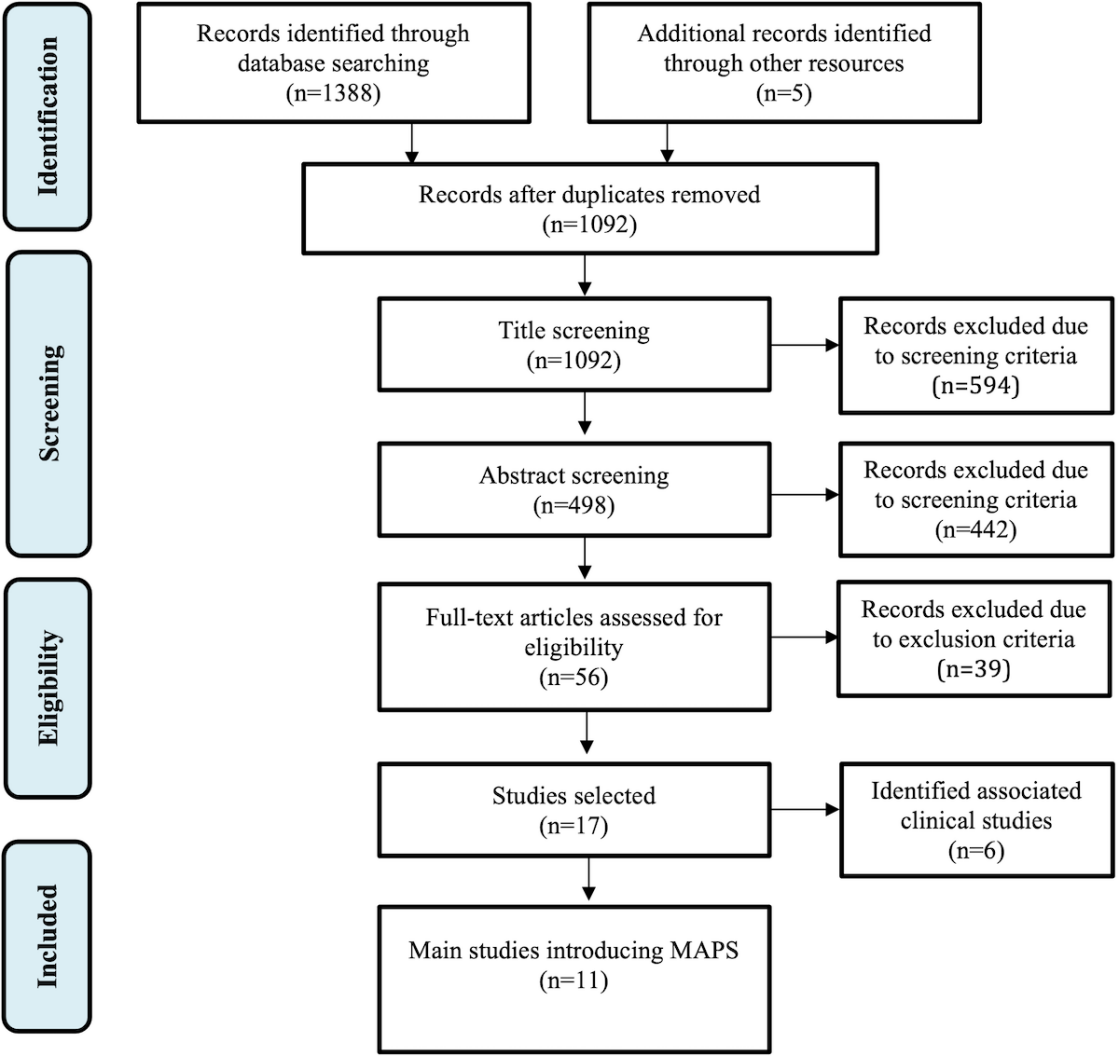
Study selection and identification flowchart. MAPS: multi-input artificial pancreas systems.

The literature survey was conducted according to the PRISMA (Preferred Reporting Items for Systematic Reviews and Meta-Analyses) framework [34]. We searched Scopus and IEEE Xplore (to capture engineering studies on APS development, including multiple-input scenarios) and PubMed (to capture APS clinical studies conducted corresponding to the identified engineering approaches) databases between January 1, 2005, and February 10, 2020. The survey focused on analyzing and summarizing the different input sources, in addition to glucose level measurements, integrated into MAPS; control algorithms; architectures; and the validation methodologies used. The clinical transition of the identified studies was also considered to obtain a complete picture of the current state of progress of MAPS developments. The study selection process was carried out in 4 steps (Figure 2).

### Identification Phase

A broad search query (Table 1) was developed to identify all papers related to control of the APS. The search query was not restricted further to ensure that all studies related to MAPS were captured. For the Scopus database, the search was restricted to articles and conference proceedings related to the subject areas of engineering, computer science, mathematics, decision sciences, and multidisciplinary specializations. The subject area restriction was not possible in PubMed; thus, the query was adjusted to exclude review articles and only include research related to humans. No additional filtering was carried out in the IEEE Xplore database because of it specific focus on computer science and electrical engineering. Additional records were identified through following the references in the selected studies (Multimedia Appendix 1).

**Table 1:** Search queries resulting in the identified studies (N=1388).

Database	Search strategy	Studies, n (%)
Scopus	(((close* AND *loop*) AND (diabet* OR *t1d*)) OR *artificial W/2* *pancreas*) AND (control*); (filter: subject area and article type)	668 (48.12)
PubMed	(((close* AND *loop*) AND (diabet* OR *t1d*)) OR *artificial W/2* *pancreas*) AND (control*) NOT (review*); (filter: humans)	393 (28.31)
IEEE Xplore	(((close* AND *loop*) AND (diabet* OR *t1d*)) OR *artificial pancreas*) AND control*	327 (23.56)

### Screening Phase

Papers identified from the database search and other resources were first analyzed to remove duplicates. The titles and abstracts of the remaining papers were screened, where papers focusing on animal studies; CGM sensor development and errors analysis; insulin pumps; other aspects of the APS (eg, safety, user experience, and psychosocial aspects); physiological modeling; studies related to the chemical, biological, and medical aspects of APS design; studies focusing on developing submodules for the AP (eg, glucose-level estimation and meal detection); studies without additional input signals; and other irrelevant studies were excluded.

### Eligibility Phase

The remaining full papers were analyzed and included in the study if the following selection criteria were met: (1) a control algorithm or architecture is present, (2) external additional inputs are used for the controller design (ie, in addition to CGM measurements, and the additional inputs are not control inputs, such as the coinfusion of glucagon), and (3) a validation is conducted in silico or in vivo (in humans). These criteria were formulated to encompass the 3 main verticals of the survey to understand and summarize the use of additional wearables in AP design, AP development technologies, and validation methodologies.

### Inclusion Phase

Finally, the selected studies (N=17) were categorized into two groups: studies that introduce different unique MAPS (11/17, 65%) and their associated clinical studies (6/17, 35%). It is important to note that some of the studies in the first category also included clinical trial results (3/11, 27%). This separation was required to avoid the duplication of similar APS and ensure the overall analysis of the identified technical criteria of the unique MAPS studies. The main studies were analyzed based on the additional inputs used, APS controller design, and validation methodologies. The clinical studies were analyzed to discuss the feasibility of MAPS.

### Quality Assessment

A quality assessment of the selected 17 studies were carried out using the Critical Appraisal Skills Programme Tool [35] (Multimedia Appendix 2 [36-52]). It is important to note that the main issues highlighted by the assessment were (1) difficulty in ascertaining the risk of bias in data collection or simulation data and (2) the failure of the study reports to provide sufficient information regarding ethical approvals.

## Results

### Results Overview

The analysis first identified the research groups working in the area of MAPS based on the selected studies and their clinical trials. Next, the shortlisted studies were evaluated based on the following main dimensions: (1) the types of additional signals used and their impact on glucose regulation, (2) the control algorithms and architectures, and (3) the validation methodologies.

### Research Groups Focusing on MAPS

The Illinois Institute of Technology and Oregon Health & Science University were identified as the 2 main research groups developing MAPS, having produced 45% (5/11) of the main studies and 83% (5/6) of the associated clinical studies (Table 2). The diversity of researchers from different domains authoring the selected studies highlights the importance of multidisciplinary teams in APS development.

**Table 2:** Breakdown of main research groups focusing on developing multi-input artificial pancreas systems (N=17)^a^.

Research group	Selected main studies (n=11), n (%)	Associated clinical studies (n=6), n (%)
Illinois Institute of Technology, United States Department of Chemical and Biological Engineering Department of Biomedical Engineering Department of Biobehavioral Health Science Department of Pediatrics Department of Electrical and Computer Engineering University of Illinois Chicago, United States College of Nursing University of Chicago, United States Biological Sciences Division Department of Pediatrics and Medicine, Kovler Diabetes Center Michigan State University, United States Sparrow Medical Group	3 (27) [36-38]	3 (50) [39-41]
Oregon Health & Science University, United States Department of Biomedical Engineering Department of Medicine Division of Endocrinology, Harold Schnitzer Diabetes Health Center Oregon Clinical and Translational Research Institute Biostatistics & Design Program Department of Medicine, Division of Health Promotion and Sports Medicine	2 (18) [42,43]	2 (33) [44,45]
Instituto Potosino de Investigación Científica y Tecnológica, Mexico Division de Matematicas Alicadas Biodinamica y Sistemas Alineales	2 (18) [46,47]	—^b^
National University of Sciences & Technology, Pakistan Department of Electrical Engineering Northwestern Polytechnical University, China School of Automation Center for Emerging Sciences Engineering and Technology, Pakistan Department of Electronics Engineering	2 (18) [48,49]	—
Stanford University, United States Division of Pediatric Endocrinology Rensselaer Polytechnic Institute, United States Department of Chemical and Biological Engineering	1 (9) [50]	—
University of Virginia, Charlottesville, Virginia, United States Center for Diabetes Technology, Division of Pediatric Endocrinology, Department of Pediatrics Division of Endocrinology, Department of Medicine Virginia Commonwealth University Division of Pediatric Endocrinology, Department of Pediatrics	1 (9) [51]	1 (17) [52]

^a^The selected 11 studies and their corresponding 6 clinical trials are categorized according to their main institutions.

^b^No associated clinical studies identified through literature search.

### Noninvasive Inputs

The types of additional inputs integrated or proposed to be integrated into APS that were identified can be categorized as (1) noninvasive inputs captured through wearable devices (most of them) and (2) invasive inputs of substances measured in body fluids. Most (9/11, 82%) of the selected studies focused on using noninvasive wearable input for MAPS development. Electrocardiogram (ECG), HR, accelerometers, skin resistance, energy expenditure (EE), and galvanic skin response (GSR) were identified as the noninvasive sensory inputs, and clinical studies were carried out for all these additional inputs, except for ECG for which simulations were conducted. However, it should be noted that wearable devices capable of capturing ECG measurements are currently available but might not have been available at the time the respective studies were carried out. Readers are directed to the study by Iqbal et al [53], which summarizes wearable devices in health care.

The additional inputs were introduced to the APS to address the previously explained limitations such as meals, exercise, stress detection, and illnesses and to counter the delays associated with glucose sensing and insulin action. It is important to analyze what additional signals have been used to counter these limitations and how they have been used in the APS design. A large portion of the studies (7/11, 64%) focused on exercise detection. They mainly used HR, accelerometer, and EE (also referred to as metabolic equivalent [MET]) for exercise detection.

Turksoy et al [36,37] and Hajizadeh et al [38] used the readily available EE data from wearables, whereas Jacobs et al [42] and Resalat et al [43] used a regression model introduced by Zakeri et al [54] to convert HR and accelerometer data to calculate MET. Stenerson et al [50] used HR and accelerometer data, and DeBoer et al [51] used HR data to identify exercise through predefined threshold values. It can be identified that exercise detection was the main focus of previous studies because of its practical importance.

Hypoglycemia prediction, using additional physiological signals, was the next popular approach to MAPS design. Predicting hypoglycemia in advance helps mitigate the glucose-sensing delays. Khan et al [48] and Qaisar et al [49] used HR, ECG (QT interval), and skin resistance for hypoglycemia detection. They identified the QT interval as the most prominent input and skin resistance as the least important input in hypoglycemia prediction. Turksoy et al [55] used EE and GSR to develop a module for hypoglycemia prediction. Stress detection was identified as another important aspect for MAPS design, where Turksoy et al [36,37] focused on using GSR signals. Patek [31], in his review, discusses a variety of other potential examples of how wearable sensory inputs can be used for MAPS design. They include the use of step counts, GPS, electroencephalography, chewing detection, finger and arm motion detection, and sleep detection data.

Managing meal effects is vital in APS development and at present it is challenging because of the heavy user burden, inaccuracies in carbohydrate counting, and forgetting to bolus. Turksoy et al [56,57] developed meal detection and carbohydrate estimation algorithms based on CGM measurements. However, in this survey, our focus was specifically on the use of MAPS design. Additional signals explored in the analyzed studies were not specifically used to improve glucose regulation related to meals.

### Invasive Inputs

People with T1D who choose not to conduct multiple daily blood glucose level tests and use multiple daily injections are currently compelled to use minimally invasive CGM and CSII devices. This requires users to take necessary steps to regularly change the sensors [26,58-61]. Hence, an additional invasive sensor might be identified as practically burdensome. However, there exists the possibility of integrating additional sensors in currently used devices such as CGM and CSII to avoid additional user burden. Previous studies have identified relationships between invasive inputs and T1D (eg, ketone sensors to identify diabetic ketoacidosis [62]). Although rich relationships exist, progress is stunted because of the lack of sensors for carrying out continuous measurements. At present, real-time interstitial insulin sensors and ketone sensors are being developed [63].

Quiroz and Femat [46] and Quiroz et al [47] identified lactate and adrenaline as 2 important invasive inputs that were directly integrated as inputs in the controller. They are used in detecting exercise and hypoglycemia, respectively, which are important aspects in APS design to address the limitations identified previously. The studies described did not focus on clinical trials based on these additional invasive inputs, which again highlights the limited research conducted in the area because of the bottleneck in sensor development.

### MAPS Architectures

The additional inputs identified in the previous section have been integrated into different architectures to augment the controllers (Table 3). They have been (1) used to switch controller modes through activity detection, (2) directly incorporated in the controller for decision-making, and (3) used for the development of intermediate modules for the controller (eg, hypoglycemia and meal detection).

**Table 3:** Summary of selected studies. Additional summarization is provided in Multimedia Appendix 3 [38,42,43,46-50].

Study	Additional inputs	Control algorithm	Architecture	Validation
Quiroz et al [46]	Lactate and adrenaline	*H*_∞_ controller	Additional inputs directly integrated	MATLAB simulation
Quiroz et al [47]	Lactate and adrenaline	*H*_∞_ controller	Additional inputs directly integrated	MATLAB simulation
Khan et al [48]	ECG^a^, HR^b^, and skin resistance	PID^c^ controller	Fuzzy fusion controller to fuse the additional input to prompt glucagon infusion (dual hormone)	MATLAB simulation
Qaisar et al [49]	ECG, HR, and skin resistance	Neural network predictive controller	Fuzzy fusion controller to fuse the additional input to prompt glucagon infusion (dual hormone)	MATLAB simulation
Stenerson et al [50]	HR and accelerometer	PLGS^d^ algorithm	Additional inputs used to switch between modes	Simulator (not specified)
DeBoer et al [51]	HR	Control to range	Additional inputs used to switch between modes (only basal rate is controlled)	Clinical study
Jacobs et al [42]	EE^e^ (HR and accelerometer used to calculate)	FMPD^f^ controller	Additional inputs used to switch the controller to a different mode (dual hormone)	Simulation; clinical study
Resalat et al [43]	MET^g^ (HR and accelerometer)	Adaptive run-to-run MPC^h^	Inputs used to calculate MET, which is directly used by the controller for decision-making; meal data also provided to the controller	Simulation
Turksoy et al [36]	EE and GSR^i^	GPC^j^	Additional inputs integrated directly; ARMAX^k^, recursive least squares, and constrained optimization used	Clinical study
Turksoy et al [37]	EE and GSR	GPC	Additional inputs integrated directly; time-varying forgetting factor for WRLS^l^ algorithm and trajectory tracking	Clinical study
Hajizadeh et al [38]	EE (MET)	Adaptive MPC	Additional inputs integrated directly into the controller. Recursive subspace identification techniques, PIC^m^, and meal estimates also used as inputs to the controller	Simulation

^a^ECG: electrocardiogram.

^b^HR: heart rate.

^c^PID: proportional integral derivative.

^d^PLGS: predictive low-glucose suspend.

^e^EE: energy expenditure.

^f^FMPD: fading memory proportional derivative.

^g^MET: metabolic equivalent.

^h^MPC: model predictive control.

^i^GSR: galvanic skin response.

^j^GPC: generalized predictive control.

^k^ARMAX: autoregressive moving average with external input.

^l^WRLS: weighted recursive least squares.

^m^PIC: plasma insulin concentration.

Stenerson et al [50], DeBoer et al [51], and Jacobs et al [42] focused on switching the mode of the controller based on detected activity. HR and accelerometer input were used in this approach, where the controller mode was changed through adjusting parameters and thresholds within the controller. Stenerson et al [50] suspended their predictive low-glucose suspend algorithm, and DeBoer et al [51] adjusted the hypoglycemia risk threshold in their control-to-range controller when exercise was detected. Jacobs et al [42] used a fading memory proportional derivative dual hormone controller that, upon the detection of exercise, carried out dosing of insulin and glucagon based on a set of static rules. This approach only focused on activity detection. However, the identified additional inputs may contain valuable information related to the glucose regulation process. Hence, studies have focused on direct integration of the additional inputs for decision-making.

Quiroz and Femat [46], Quiroz et al [47], Resalat et al [43], Turksoy et al [36,37], and Hajizadeh et al [38] focused on direct integration of additional inputs in the controller design. Quiroz and Femat [46] and Quiroz et al [47] directly integrated lactate and adrenaline input into their *H*_∞_ control algorithm. Resalat et al [43] developed a run-to-run MPC that used continuous MET data for exercise detection. Turksoy et al [36] integrated EE and GSR into a generalized predictive controller by developing time series models using autoregressive moving average with external input, recursive least squares, and constrained optimization techniques. They improved on their work by introducing a time-varying forgetting factor for the weighted recursive least squares algorithm and focusing on trajectory tracking [37]. Hajizadeh et al [38] used recursive subspace identification techniques to develop an adaptive MPC controller incorporating MET input. The continuously integrated inputs such as EE and GSR provided valuable and timely insights regarding the glucose regulatory process, which is valuable.

Designing submodules for the APS has also been widely explored, where the focus has been on using the input to enhance insulin and glucagon infusion and to design safety mechanisms for the APS. These submodules were mainly linked to identified limitations such as meal detection, activity detection, and hypoglycemia detection. Khan et al [48] and Qaisar et al [49] developed a hypoglycemia-detection module using HR, ECG (QT Interval), and skin resistance. In addition to the main controller focusing on insulin infusion, a fuzzy logic fusion controller was introduced to infuse glucagon based on the identified signals during a hypoglycemia event. Turksoy et al [41] performed a clinical trial where hypoglycemia early alarm [55], meal detection [56,64], hypoglycemia prediction, and carbohydrate recommendation [57] modules were integrated into the final APS design. Hajizadeh at al [38] focused on plasma insulin concentration estimation and meal effect estimation modules in their research. Resalat et al [43] proposed and evaluated an insulin sensitivity adaptation algorithm and an adaptive-learning postprandial hypoglycemia prevention algorithm. However, it is important to note that some of these submodules only used existing CGM measurements. Different safety modules have also been introduced, where Turksoy et al [36] and DeBoer et al [51] focused on hypoglycemia and hyperglycemia safety, respectively, through insulin-on-board estimates. The development of submodules enhances the interpretability of the APS operation, which is essential in safety-critical applications. Most of the studies have used submodules in their controllers, both with switching the controller mode through activity detection and when additional inputs are directly integrated. Hence, designing submodules using additional input targeting the identified limitations is beneficial in APS development.

### Validation Methodologies

The designed APS have been validated through simulations and clinical studies (Tables 3 and 4). A variety of physiological models and tools have been used for simulations and different protocols used for clinical trials. The AP is classified as a high-risk medical device by the FDA, which requires proper simulation and testing before conducting clinical trials. However, it is important to note that an FDA-approved simulator is currently unavailable for testing MAPS. In all, 2 groups have focused on developing their own multiple-input simulators [24,25], which would be beneficial for the progress of MAPS development.

**Table 4:** Comparison of clinical trial results.

Author	Trial and controller setting	Results
Breton et al [52]	12 adults, randomized crossover trial, 24-hour closed-loop experiments each with exercise Exercise detection using HR^a^ Meal bolus manually calculated	Time in euglycemia^b^ for AP^c^ with HR and without HR overall 81% vs 75%, exercise 91% vs 85%, and overnight 89% vs 84% Using HR resulted in fewer hypoglycemic events during exercise (0 vs 2)
DeBoer et al [51]	18 adolescents, randomized crossover trial, 24-hour closed-loop experiments each with exercise Exercise detection using HR Meal bolus manually calculated	Time in euglycemia for AP with HR and without HR overall 77% vs 74%, exercise 96% vs 87%, and overnight 92% vs 84% Small reduction in hypoglycemic events (0.39 HR-informed AP vs 0.50 without HR)
Jacobs et al [44]	21 adults, randomized crossover trial, 22-hour experiments each with exercise Exercise-detection algorithm triggered manually	Time in euglycemia with exercise detection 67%, without exercise detection 72%, and SAP^d^ 68% Time in hypoglycemia (<3.9 mmol/L) 0.3%, 3.1%, and 0.8%, respectively Time in hyperglycemia (<10 mmol/L) 32%, 25%, and 31%, respectively
Castle et al [45]	20 adults, randomized crossover trial, 4-day experiments each with exercise Exercise-detection algorithm triggered using wearable sensor in SH^e^ and DH^f^ controllers	Time in euglycemia overall SH 74.3%, DH 72%, PLGS^g^ 65.2%, and current care 63.1% Time in hypoglycemia 2.8%, 1.3%, 2%, and 3.1%, respectively
Turksoy et al [36,39]	3 young adults, seven 32- or 60-hour closed-loop experiments with exercise Additional signals integrated continuously	Time in euglycemia 62% (overnight 75.3%, exercise 55%, and glycemic closed loop 56.1%)
Turksoy et al [37]	3 young adults, 70-hour closed-loop experiments with exercise Additional signals integrated continuously	Time in euglycemia 46.5%
Turksoy et al [40]	9 young adults, 2-day closed-loop experiments with exercise Additional signals integrated continuously	Time in euglycemia 58%
Turksoy et al [41]	10 young adults, eighteen 60-hour closed-loop experiments with exercise Additional signals integrated continuously, with submodules	Time in euglycemia 69.9% for exercise and recovery periods and 76.75% overall performance

^a^HR: heart rate.

^b^Euglycemia target range 70-180 mg/dL (Jacobs et al [44] report euglycemia as 3.9-10 mmol/L, range 70.2-180 mg/dL, whereas all other studies report results for the range 70-180 mg/dL).

^c^AP: artificial pancreas.

^d^SAP: sensor-augmented pump.

^e^SH: single hormone.

^f^DH: dual hormone.

^g^PLGS: predictive low-glucose suspend.

MATLAB was used in most of the studies to conduct simulations. Quiroz et al [46,47] simulated the use of invasive inputs based on the Sorenson model [21], the Bergman minimal model [65], the glucose–adrenaline relationship discussed in the study by Schultes et al [66], and the glucose–lactate relationship discussed in the study by Stuart et al [67]. Khan et al [48] and Qaisar et al [49] also used the Bergman minimal model, as well as simulated meals, ECG, and subcutaneous delays. Jacobs et al [42] used the Hovorka insulin pharmacodynamics model [68], the insulin pharmacokinetics model by Wilinska et al [69], the glucagon pharmacokinetics model by Lv et al [70], the glucagon pharmacodynamics model by Bakhtiani et al [71], and the exercise model by Hernandez-Ordonez et al [72] for their simulation. Resalat et al [43] and Hajizadeh et al [38] conducted their simulations based on simulators developed by their own research groups [24,25].

DeBoer et al [51], Breton et al [52], and Jacobs et al [44,45] carried out a clinical trial to evaluate their switching mode controller after obtaining FDA and institutional review board approvals. Breton et al [52] and DeBoer et al [51] reported a reduction in hypoglycemic events in adolescents and adults, respectively, using HR in an activity-augmented control architecture. Jacobs et al [44] also achieved a reduction in time spent in hypoglycemia, but there was an increase in the time spent in hyperglycemia when the exercise-augmented control structure was used. Similar results were observed in the subsequent trial by Castle et al [45]. Overall, these randomized crossover trials were able to identify a reduction in hypoglycemia when the activity-augmented control structure was used. It is important to note that activity-augmented APS design might be compromised during different types of exercises (high-intensity training and resistance exercise), which has not been explored. Turksoy et al [39-41] focused on having a medical expert to review each insulin dose before the application and obtained institutional review board approval. They focused on integrating continuous inputs (EE and GSR) into the controller and developing submodules and conducted clinical trials for evaluation. They succeeded in improving the time in target range (70-180 mg/dL) to 76.75% with the integration of different submodules into the APS. The identified clinical trials (Table 4) focused on either adolescents, young adults, or adults. The trials comprised both normal closed-loop trials and randomized crossover trials, which evaluated different treatment types and typically ranged in duration from 1 to 4 days. Further longitudinal studies will be beneficial to ascertain the effects of sensor noise and unanticipated dropouts that might arise from the additionally introduced sensors. It is important to conduct trials encompassing all age groups (children, adolescents, and adults) to evaluate the robustness of the controllers because different age groups have different insulin sensitivities, which affects the controller’s accuracy.

## Discussion

### Principal Findings

This survey focused on three main verticals: (1) identifying the types of additional input signals used, (2) analyzing different APS control methodologies, and (3) exploring MAPS validation methodologies. In this section, a summary of the findings based on these aspects, a discussion on the feasibility of MAPS, a comparison of clinical trial results, and limitations of the conducted survey are discussed.

**Figure 3:**
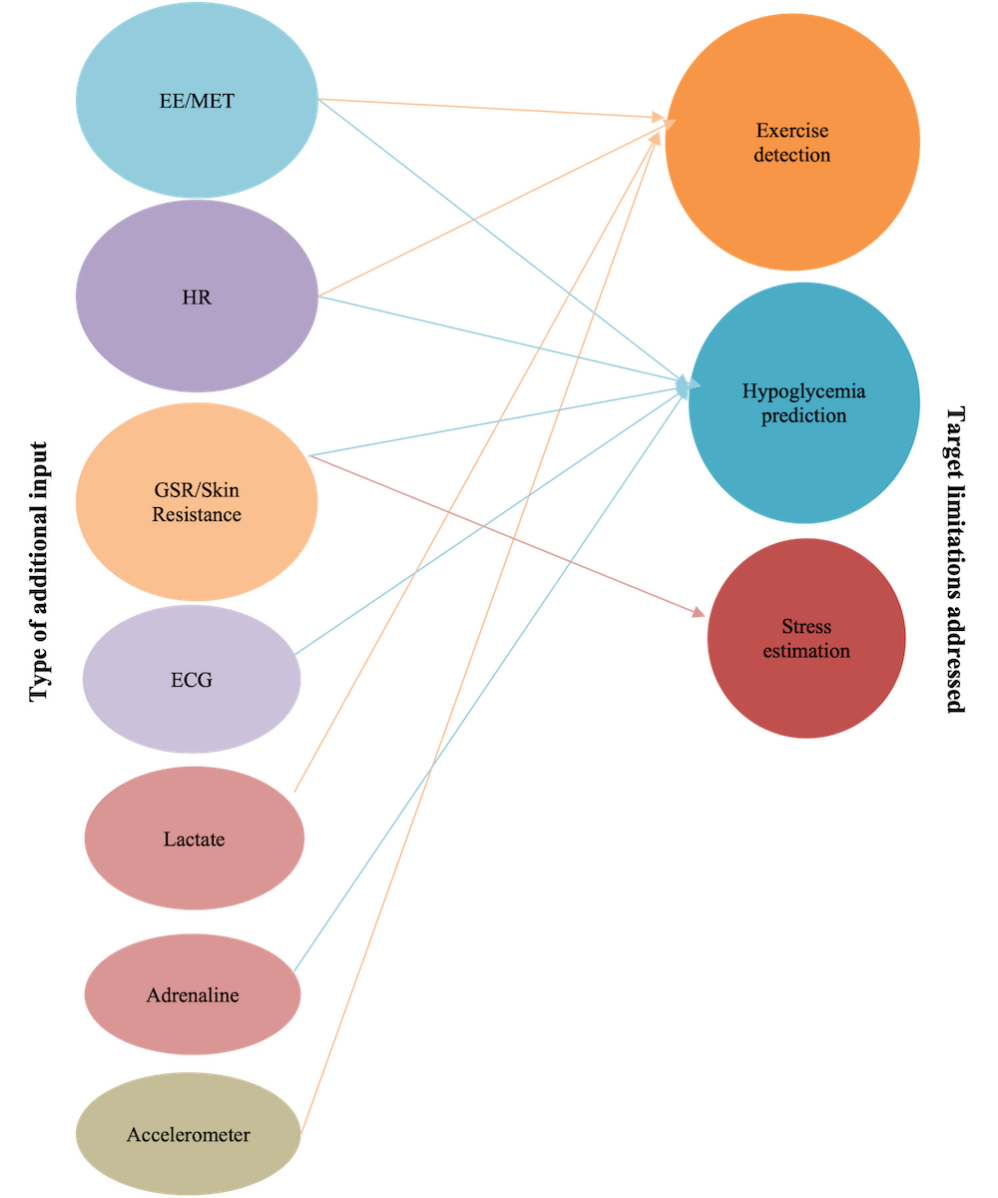
Distribution of additional inputs used in the final artificial pancreas systems design and their main focus aspects. Only the additional inputs used in the final design are presented. Input variables used to synthesize the final inputs have been removed. ECG: electrocardiogram; EE: energy expenditure; HR: heart rate; GSR: galvanic skin response; MET: metabolic equivalent.

Most of the identified inputs were noninvasive, captured through wearable devices. However, the effectiveness of invasive inputs has also been analyzed through simulations. Lactate and adrenaline were the identified invasive inputs used for exercise detection and hypoglycemia detection. EE (or MET) can be identified as the most frequent additional input used in APS development. EE is able to detect exercise, which helps mitigate the related APS limitations identified previously. Hypoglycemia prediction has been carried out through the use of inputs such as ECG, HR, skin resistance, EE, and GSR. GSR has also been used effectively as an indicator of stress. HR-, EE-, GSR-, and accelerometer-based studies have been evaluated through clinical trials mainly because of the easy access through wearable devices. The technological advancements in wearable devices would be beneficial for the development of MAPS. A summary of the distribution of different additional inputs used in the final APS design and their main focus aspects in the selected studies is provided in Figure 3.

Most of the studies (8/11, 73%) focused on augmenting single-hormone APS compared with dual-hormone systems. Identifying additional inputs that can be used to address current limitations and directly integrating those inputs into the controller has shown promise. The development of submodules based on these limitations and switching the mode of the controller through activity detection can also be identified as effective approaches to MAPS design. Different control algorithms and architecture have been proposed in previous research. Adaptive model–based controlling methods have been frequently used for controller development.

Validations were carried out in the studies in silico (7/11, 63%) as well as in vivo (4/11, 36%). Both quantitative and qualitative metrics were used to evaluate the effectiveness of the proposed systems. The time in hypoglycemia, euglycemia, and hyperglycemia ranges as well as the number of hypoglycemic events were some of these measures. However, comparison of the results is subjective because of the different physiological models used in the simulators and different protocols (exercise, meals, and age groups) used in the clinical studies. Furthermore, some of the studies included additional modules such as hypoglycemia alarms and meal detection, which were unrelated to the analyzed additional inputs in this study. This further limited a valuable interstudy statistical analysis to understand the impact of the proposed additional Inputs. However, an analysis of comparable studies within the same research group has been presented in the previous section. It is important to mention that 2 groups had focused on developing their own simulators [24,25] because currently available simulators did not have other multiple inputs incorporated. The rest of the studies had combined different physiological models in previous research to simulate the additional variables. At present, such a validated simulator is yet to be developed for MAPS. The development of an FDA-approved simulator for MAPS would be beneficial to test and compare different proposed control architectures to statistically evaluate their performance and the progress in this area. The studies analyzed in the survey have obtained FDA and institutional review board approvals to conduct clinical trials.

It is important to review the patents published related to APS to identify possible technological advancements. We conducted a search on Google Patents for the period January 2005 to May 2021 and identified 2 patents associated with MAPS (Multimedia Appendix 4 [73,74]). Both the patents were associated with the Illinois Institute of Technology research group identified in the previous section. Patent ID US8690820B2 [73] presented a device where a glucose sensor and physiological status–monitoring system communicate with an automatic controller for glucose control. The controller also included a module to predict future glucose levels. Patent ID US10646650B2 [74] introduced additional modules for recursive model identification of hypoglycemia and hyperglycemia early alert and alarm, plasma insulin concentration estimation, physical activity assessment, stress detection and assessment, sleep detection, and sensor and pump fault detection and diagnosis. The aforementioned proposed modules using physiological signals were identified in the previous section.

### Feasibility of MAPS

Different additional inputs have been identified and used to address limitations identified in current generation APS. However, more signals and relationships need to be explored to address limitations such as meal and illness estimation. It is important to quantify the improvement of the APS through the integration of additional input signals. The benefits should outweigh the burden of using the external sensors.

The results of the proposed approaches can be analyzed based on their clinical trials, which provides a fairer interpretation compared with the simulations. However, it should be noted that comparison between trials is not straightforward because of the different protocols (meals and exercise) and the number of participants involved. The identified clinical trials improved the time in euglycemia range and showed a reduction in hypoglycemic events when additional inputs were used. However, further trials need to be conducted with larger cohorts and trial durations to ensure the effectiveness of the systems.

The noise and instability associated with wearable sensors also need to be evaluated because they could have a detrimental effect on the controllers. Precautionary measures should be set in place to ensure patient safety during such circumstances. It is also important to note that the real-world application of MAPS would be very complex. For example, a person with T1D might not wear additional wearables during sleep, which might require the controller to work in highly dynamic environments. Hence, it is important to evaluate such scenarios through simulations and clinical studies conducted for longer durations.

### Conclusions and Comparison With Prior Work

Kudva et al [30] analyzed the clinical importance of incorporating additional signals, and Cinar [29] and Patek [31] analyzed the current limitations in APS design and the approach to MAPS development. In this survey, we analyzed existing APS designs to identify the types of input variables used, control techniques, architectures, and validation methodologies. This survey was restricted to studies that proposed APS. However, research studies exist that aim to identify relationships between various physiological signals and T1D. The identification of such relationships would be beneficial for the development of MAPS. Previous research has also focused on designing submodules such as meal detection [56], carbohydrate recommendation [57], and hypoglycemia prediction [55] modules for APS. Given the scope of this survey, such submodules were only identified and only the final integrated APS were evaluated. This survey mainly focused on the technical aspects of MAPS development. It is also important to explore and evaluate the corresponding practical aspects (eg, additional user burden, sensor failures, and psychosocial impact).

The integration of additional signals is an approach to mitigate the current limitations of the APS. Most of the integrated additional inputs in previous research are from wearables. The widespread availability of wearables could be seen as a factor facilitating MAPS. Past studies have mainly focused on using the additional inputs for detecting exercise (HR, accelerometer, and EE), hypoglycemia (ECG, HR, EE, and GSR), and stress (GSR). In future, these additional sensors might also be valuable in capturing other physiological changes such as illnesses, alcohol consumption, and seasonal variations. Previous randomized crossover studies were able to obtain lower time in hypoglycemia and improvements in the normal glycemic range when additional inputs were integrated. However, these systems need to be improved to obtain better time in target range for glucose to improve the quality of life of people with T1D. The lack of an FDA-approved simulator for testing the identified additional input can be identified as a major constraint regarding the development of MAPS. It is important to explore different additional inputs further to establish relationships with glucose regulation and use them to address the identified limitations. The practical complexities and psychosocial aspects associated with MAPS need to be evaluated to develop effective APS.

## Appendix

Multimedia Appendix 1 Search query formulation.

Multimedia Appendix 2 Quality assessment.

Multimedia Appendix 3 Summary of additional inputs and simulation models.

Multimedia Appendix 4 Patents associated with multi-input artificial pancreas systems.

## Abbreviations

ANU: Australian National University
AP: artificial pancreas
APS: artificial pancreas systems
CGM: continuous glucose monitoring
CSII: continuous subcutaneous infusion of insulin
ECG: electrocardiogram
EE: energy expenditure
FDA: Food and Drug Administration
GSR: galvanic skin response
HR: heart rate
MAPS: multi-input artificial pancreas systems
MET: metabolic equivalent
MPC: model predictive control
PRISMA: Preferred Reporting Items for Systematic Reviews and Meta-Analyses
T1D: type 1 diabetes
