# Supplementary material for: Integrating Multiple Inputs Into an Artificial Pancreas System: Narrative Literature Review
Source: JMIR Diabetes. 2022 Feb 24;7(1):e28861. doi: 10.2196/28861 (PMC8914747; doi:10.2196/28861)
Supplement: Multimedia Appendix 1 [file diabetes_v7i1e28861_app1.docx]

**Multimedia Appendix 1**

**Search Query Formulation**

Different strategies were explored to develop a suitable search query with the help of a research librarian. It was identified that the studies were not able to be simply queried through the use of the Medical Subject Headings (MeSH) terms due to the following constraints:

- All papers had not been indexed using MeSH terms (e.g., diabetes mellitus, type 1 and pancreas, artificial).
- Some MeSH terms (e.g., Devices, Wearables) had been introduced recently whereas this survey focused on literature for the period 2005–2020.

Hence, the proposed query (Table 1) was designed to encompass all studies related to APS and control. The queries were not restricted by terms, such as “wearables, physiological signals”, in order to ensure that none of the potential studies was omitted. Hence, instead all the 1388 results from the general queries were manually screened to identify the appropriate studies.
