# Supplementary material for: Integrating Multiple Inputs Into an Artificial Pancreas System: Narrative Literature Review
Source: JMIR Diabetes. 2022 Feb 24;7(1):e28861. doi: 10.2196/28861 (PMC8914747; doi:10.2196/28861)
Supplement: Multimedia Appendix 2 [file diabetes_v7i1e28861_app2.docx]

**Multimedia Appendix 2**

**Quality Assessment**

| CASP QUESTIONS  Question 1: Was there a clear statement of the aims of the research?  Question 2: Is a qualitative methodology appropriate?  Question 3: Was the research design appropriate to address the aims of the research?  Question 4: Was the recruitment strategy appropriate to the aims of the research?  Question 5: Was the data collected in a way that addressed the research issue?  Question 6: Has the relationship between researcher and participants been adequately considered?  Question 7: Have ethical issues been taken into consideration?  Question 8: Was the data analysis sufficiently rigorous?  Question 9: Is there a clear statement of findings?  Question 10: How valuable is the research?  Y = YES N = NO CT = CAN’T TELL NA = NOT APPLICABLE | | | | | | | | | | |
| --- | --- | --- | --- | --- | --- | --- | --- | --- | --- | --- |
| Author, Year | Q1 | Q2 | Q3 | Q4 | Q5 | Q6 | Q7 | Q8 | Q9 | Q10 |
|  |  |  |  |  |  |  |  |  |  |  |
| **Main Studies** |  |  |  |  |  |  |  |  |  |  |
|  |  |  |  |  |  |  |  |  |  |  |
| Quiroz et al 2010 [46] | Y | Y | Y | NA | NA | CT | NA | Y | Y | Y |
| Quiroz et al 2011 [47] | Y | Y | Y | NA | NA | CT | NA | Y | Y | Y |
| Khan et al 2013 [48] | Y | Y | Y | NA | NA | CT | NA | Y | Y | Y |
| Qaisar et al 2012 [49] | Y | Y | Y | NA | NA | CT | NA | Y | Y | Y |
| Stenerson et al 2014 [50] | Y | Y | Y | Y | Y | N | Y | Y | Y | Y |
| DeBoer et al 2017 [51] | Y | Y | Y | Y | Y | CT | Y | Y | Y | Y |
| Jacobs et al 2015 [42] | Y | Y | Y | Y | Y | CT | CT | Y | Y | Y |
| Reslat et al 2019 [43] | Y | Y | Y | Y | Y | CT | CT | Y | Y | Y |
| Turksoy et al 2013a [36] | Y | Y | Y | Y | Y | CT | N | Y | Y | Y |
| Turksoy et al 2013b [37] | Y | Y | Y | Y | Y | CT | CT | Y | Y | Y |
| Hajizadeh et al 2019 [38] | Y | Y | Y | NA | NA | CT | NA | Y | Y | Y |
|  |  |  |  |  |  |  |  |  |  |  |
| **Clinical Studies** |  |  |  |  |  |  |  |  |  |  |
|  |  |  |  |  |  |  |  |  |  |  |
| Breton et al 2014 [52] | Y | Y | Y | Y | Y | CT | Y | Y | Y | Y |
| Jacobs et al 2016 [44] | Y | Y | Y | Y | Y | CT | Y | Y | Y | Y |
| Castle et al 2018 [45] | Y | Y | Y | Y | Y | CT | Y | Y | Y | Y |
| Turksoy et al 2013a [39] | Y | Y | Y | Y | Y | CT | CT | Y | Y | Y |
| Turksoy et al 2014 [40] | Y | Y | Y | Y | Y | CT | Y | Y | Y | Y |
| Turksoy et al 2018 [41] | Y | Y | Y | Y | Y | CT | Y | Y | Y | Y |
