# Supplementary material for: Integrating Multiple Inputs Into an Artificial Pancreas System: Narrative Literature Review
Source: JMIR Diabetes. 2022 Feb 24;7(1):e28861. doi: 10.2196/28861 (PMC8914747; doi:10.2196/28861)
Supplement: Multimedia Appendix 3 [file diabetes_v7i1e28861_app3.docx]

**Multimedia Appendix 3**

**Summary of Additional Inputs & Simulation Models.**

| **Type of additional Input** |  |
| --- | --- |
|  |  |
| Invasive | Lactate, Adrenaline |
| Non-Invasive Wearable | Heart Rate, Accelerometer, ECG, Skin Resistance, Energy Expenditure, Galvanic Skin Response |

| Author, Year | Simulation Model |
| --- | --- |
|  |  |
| Quiroz et al 2010 [46] | Sorenson’s Model [21], Bergman’s Minimal Model [65], Glucose – adrenaline relation - Schultes et al (2007) [66], and Glucose – lactate relationship - Stuart et al (2001) [67]. |
| Quiroz et al 2011 [47] | Sorenson’s Model [20], Bergman’s Minimal Model [65], Glucose – adrenaline relation - Schultes et al (2007) [66], and Glucose – lactate relationship - Stuart et al (2001) [67]. |
| Khan et al 2013 [48] | Bergman’s Minimal Model [65]. |
| Qaisar et al 2012 [49] | Bergman’s Minimal Model [65]. |
| Stenerson et al 2014 [50] | Not Specified. |
| Jacobs et al 2015 [42] | Hovorka’s insulin pharmacodynamics model [68], insulin pharmacokinetics model by Wilinska et al [69], glucagon pharmacokinetics model by Lv et al [70], glucagon pharmacodynamics model by Bakhtiani et al [71], and exercise model by Hernandez-Ordonez et al [72] |
| Reslat et al 2019 [43] | Virtual patient population by Resalat et al (2019) [25]. |
| Hajizadeh et al 2019 [38] | mGIPsim simulator (2019) [24]. |
