# Supplementary material for: Integrating Multiple Inputs Into an Artificial Pancreas System: Narrative Literature Review
Source: JMIR Diabetes. 2022 Feb 24;7(1):e28861. doi: 10.2196/28861 (PMC8914747; doi:10.2196/28861)
Supplement: Multimedia Appendix 4 [file diabetes_v7i1e28861_app4.docx]

**Multimedia Appendix 4**

**Patents Associated with MAPS.**

| Patent ID | Patent Name | Application Granted | Inventor |
| --- | --- | --- | --- |
|  |  |  |  |
| US8690820B2 [73] | Automatic insulin pumps using recursive multivariable models and adaptive control algorithms. | 2014-04-08 | Inventor: Ali Cinar, Meriyan Oruklu  Current Assignee:  Illinois Inst of Technology |
| US10646650B2 [74] | Multivariable artificial pancreas method and system | 2020-05-12 | Inventor: Ali Cinar, Kamuran TURKSOY, Iman HAJIZADEH  Current Assignee:  Illinois Inst of Technology |
